# Anonymity versus Privacy in the Dictator Game: Revealing Donor Decisions to Recipients Does Not Substantially Impact Donor Behavior

**DOI:** 10.1371/journal.pone.0115419

**Published:** 2014-12-22

**Authors:** Jeffrey Winking

**Affiliations:** Department of Anthropology, Texas A&M University, College Station, Texas 77843-4352, United States of America; Universidad Carlos III de Madrid, Spain

## Abstract

Anonymity is often offered in economic experiments in order to eliminate observer effects and induce behavior that would be exhibited under private circumstances. However, anonymity differs from privacy in that interactants are only unaware of each others' identities, while having full knowledge of each others' actions. Such situations are rare outside the laboratory and anonymity might not meet the requirements of some participants to psychologically engage as if their actions were private. In order to explore the impact of a lack of privacy on prosocial behaviors, I expand on a study reported in Dana et al. (2006) in which recipients were left unaware of the Dictator Game and given donations as “bonuses” to their show-up fees for other tasks. In the current study, I explore whether differences between a private Dictator Game (sensu Dana et al. (2006)) and a standard anonymous one are due to a desire by dictators to avoid shame or to pursue prestige. Participants of a Dictator Game were randomly assigned to one of four categories—one in which the recipient knew of (1) any donation by an anonymous donor (including zero donations), (2) nothing at all, (3) only zero donations, and (4) and only non-zero donations. The results suggest that a lack of privacy increases the shame that selfish-acting participants experience, but that removing such a cost has only minimal effects on actual behavior.

## Introduction

Numerous studies suggest that participants in economic experiments are sensitive to the presence of observers, particularly when the task is governed by social norms [1]–[4]. Researchers, however, are often interested in how participants would engage under completely private circumstances, free from any concern for how their actions might be perceived by others. Anonymity is thus commonly offered to participants in economic experiments as a means of eliciting private behavior. Studies suggest that efforts to mimic private circumstances are at least partly effective, as participants tend to increase selfish behavior as researchers offer increasingly strong forms of anonymity [5]–[8], and decrease selfish behavior as their identities and actions are made increasingly public [9], [10]. However, it is unknown to what degree participants are still influenced by perceptions of non-privacy, even under the strongest forms of anonymity.

Accurately assessing the efficacy of anonymity in eliciting truly private behavior is important to a number of important debates, most notably that concerning the evolution and maintenance of prosociality in humans. Competing theories are largely differentiated by the importance placed on reputational motivations, which are often inferred from observer effects [11]–[13]. The fact that many participants exhibit prosocial tendencies, even under completely anonymous conditions, is often presented as evidence that humans have a general capacity for prosocial behaviors irrespective of reputational concerns [14], [15]. It is therefore important to determine the degree to which residual prosocial behaviors—those remaining even under strong forms of anonymity—are due to other-regarding tendencies that are independent of observer effects, such as warm-glow [16], social preferences [17], or other strong-reciprocity motivations [12], or to at least some participants still perceiving their actions to be non-private.

A number of studies have revealed just how sensitive participants are to the possibility of being observed [11], [18]–[20]. Subtle cues, such as images resembling a pair of observing eyes, increase the frequency (although not the amounts) of prosocial donations [19]. Natural field versions of economic experiments, in which participants are unaware that their actions are being recorded, are the only scenarios in which complete privacy can be replicated. Such studies present mixed results concerning whether the more natural forms of privacy induce self-regarding behaviors [6], [21], [22].

A major difference between the anonymity that is offered in the laboratory and the privacy it is meant to replicate is that under anonymity, only identities are concealed, while the actions themselves are often made known to interactants. Such situations are exceedingly rare outside the laboratory, particularly when considering direct prosocial actions. Indeed, it is difficult to conjure examples in which a person might be aware that some person has directly helped, or that some person has decided to refrain from directly helping, but not know the individual's identity. Private decisions about direct prosocial actions more commonly take the form of choosing between publicly providing help (i.e. the recipient is aware of the action and identity of the helper) or privately withholding help (i.e. the potential recipient is not even aware any decision has taken place). This is the case whenever an individual has some resource that he or she could share or secretly hoard. Therefore, the artificial contexts and the anonymity that are common to many economic experiments might not be psychologically salient to all participants.

A number of studies have shown that some participants are willing to pay a small fee in order to “exit” a Dictator Game, in which they are simply given the endowment (minus the fee), and the original decision of how to split the endowment is not carried out [23]–[27]. If participants are indifferent to their anonymous actions being made known to the recipient, exiting the game would be dominated by the option to play the game and donate zero (or an amount equal to or less than the fee). Dana et al. [23] also introduced a method in which recipients could be left unaware of donor actions, even when non-zero donations were made. Recipients in this “private” condition simply received a bonus along with their show-up fee for a different study; the bonus was equal to the donation provided by their paired dictator, but recipients were not told of the origin of the bonuses. Results were suggestive that donors were less likely to give a non-zero amount and give less overall, but with fewer than 25 participants for each condition, the effects did not attain significance. The present study builds upon these methods in three ways. First, this study utilizes an online community, which allows for a quadrupling of the sample size, allowing for a much more powerful testing of the effects. Secondly, the “exit” method is recreated in a natural way, which requires the participants to devise the strategy on their own, thus eliminating most experimenter-demand effects. Last, I explore the reputational motivations that underlie potential differences in donation amounts between private and anonymous conditions.

Reputational concerns can be primarily defined by an aversion to negative effects or a desire for positive effects, depending on the marginal gains (or losses) in either direction. For instance, little benefit might be derived from contributing increasing amounts to a collection plate, but failing to donate something might result in substantial reputational injury. Alternatively, an individual who refuses to offer money to a panhandler might experience minor reputational damage, but buying the panhandler dinner might greatly impact others' perceptions of this individual. The impact of failing to act is governed by the strength of a relevant norm to offer a minimal effort and the perceived social cost of being known as someone to have broken this norm. The cost is often directly experienced as shame, but can also lead to real, non-affective social consequences, such as the loss of social partners or even direct punishment [10], [27]–[29]. In the other direction, individuals who go beyond the minimal norm might experience a benefit in the form of prestige. Prestige can benefit an individual in many ways—others might be more willing to engage socially, economically or romantically with individuals with high prestige [30], [31]. The degree to which going beyond a norm influences prestige, as well as the payoff of this prestige to the altruist, likely depends on a multitude of factors and can vary from context to context and from person to person.

Informing others of one's anonymous decisions might induce participants to behave more prosocially because some still perceive a risk of experiencing shame for failing to conform to a norm or because some still perceive an opportunity to enhance their prestige (or both). The current study attempts to isolate these effects.

## Materials and Methods

### Study Design

In a between-subjects study design, participants took part as donors in one of four anonymous Dictator Game treatments (recipients also took part in the current study, but were passive participants who only received donations). In the Control treatment, participants acted as a donor in a traditional Dictator Game. Recipients were made aware that the game had been played and the amount the donor had decided to give. In the Private condition, the fact that the game was played and the amount of the donor's donation was not made known to the recipient regardless of donation amount. Recipients paired to donors who gave zero in this treatment simply received a show-up fee for completing basic surveys and were not told of the Dictator Game portion. Recipients paired to donors who gave a non-zero amount in this treatment were provided a bonus in addition to the show-up fee, without any explanation. If the revealing of donors' decisions evokes reputational concerns, donors in the Private condition are expected to give less overall than donors in the Control condition (H1).

Two additional treatments were included to explore the degree to which participants naturally “exit” the game, as well as the underlying potential motivations behind any differences between anonymous and private conditions. In the Private-Positive condition, the fact that the game had been played and the amount of the donor's donation was only made known to the recipient if the donor gave zero, while non-zero donations remained private in the manner described for the Private condition. If participants who regularly would donate zero experience shame for having such a decision revealed to the recipient, they should be motivated to donate the minimum amount in order to conceal their decision. Thus, we expect a lower frequency of zero donations and a higher frequency of minimum non-zero donations in the Private-Positive condition compared to the Control condition (H2). Note that the minimum non-zero donation is equivalent to the “exit” decision, although participants are not directly told that they have this option, eliminating cognitive experimenter demand effects [32].

Finally, the Private-Zero condition is also included to explore the degree to which an aversion to experiencing shame motivates actual donor behavior, not simply whether shame is experienced by those who donate zero. In this condition, the fact that the game had been played and the amount of the donor's donation were only made known to the recipient if the donor gave a non-zero amount, while zero donations remained private in the manner described for the Private condition. If participants respond to non-private elements because of a desire to not experience public shame for breaking a social norm against acting selfish, then a larger proportion of participants in the Private condition (H3a) and particularly in the Private-Zero condition (H3b) should donate zero compared to those in the Control. While such an effect is predicted in both the Private and Private-Zero conditions, the Private-Zero condition provides for a more sensitive test, as it is the only private option available.

If, however, reputational concerns are defined by a desire to appear magnanimous (i.e. prestige), participants in the Private condition who donate a non-zero amount are expected to donate less than those who donate a non-zero amount in the Control condition (H4a). Such an effect is also predicted in the Private-Positive condition, although this is conflated with H2. Therefore, participants who donate more than the non-zero minimum (5 cents) in the Private-Positive condition are expected to donate a smaller amount than those who donate more than the non-zero minimum in the Control condition (H4b).

### Procedure

The study was conducted utilizing Amazon Mechanical Turk (mTurk), a crowd-sourcing service provided by Amazon.com. Participants in mTurk studies are more demographically diverse than undergraduate participant pools [33] and differ along some axes of personality and attitudes [34]; however, they tend to respond truthfully [35], and results of behavioral studies using mTurk are comparable to those derived using conventional methods and participant pools [36], [37].

The study was conducted in three separate rounds, each separated by approximately two weeks, over the summer of 2014. Three rounds were used to reduce the likelihood that the nature of the study would be reported on message boards. The most common message board for this purpose, Reddit's “Hits Worth Turking For,” was monitored to ensure that this did not happen. Participants responded to a request that read “Participate in a short economic experiment, and then answer a short 3–5 min survey.” They were offered $0.10 as a show up fee. 500 participants took part in the study as Dictators. Participants were randomly assigned to one of the four conditions, taking part in only this one condition. They were given instructions specific to their condition and then had two chances to correctly describe the outcomes of three scenarios, including whether the participant would be aware of the donation. Sixty-three individuals or 12.6% of all participants were excluded for failing to meet this criterion. An additional 54 cases were excluded, as the individuals had participated in previous rounds or previous versions of this study. Participants were asked to partition an endowment of $1.00 in increments of $0.05. Although this endowment is substantially lower than that used in laboratory studies, it represents a substantial rate of return compared to other mTurk assignments. Furthermore, previous mTurk studies have found the distributions of Dictator Game donations of $1.00 endowments to be comparable to those using larger endowments [37], [38]. In previous mTurk studies, however, posters on “Hits Worth Turking For” had expressed doubt concerning whether money is actually given to recipients in such games. Therefore, the sentence, “This will really happen—another mTurk participant will actually receive the money you decide on,” was included in the instructions. After completing the Dictator Game, participants completed a short survey covering basic demographic information and an altruism battery adopted from the National Altruism Study that was included in the 2002 General Social Survey [39]. Donations were given to recipients in a separate round in the manner dictated by the condition of the donor. Methods were approved by the Texas A&M Human Subjects Protection Program and carried out under protocol IRB2013-0479.

## Results

The samples across the four conditions do not significantly differ in gender, age, or education (Table 1). Similarly, a composite prosociality score based on the sum of reported frequencies of 10 different prosocial behaviors (measured along six-point ordinal scales) did not differ across conditions (ANOVA, p = 0.364). The four groups did differ in income distribution, although income was not significantly correlated with donation amount in any of the four conditions.

**Table 1:** Sample Characteristics.

	N	Male	Mean Age (SD)	Median Household Income	Median Education	Mean Altruism Index (SD)
Control	98	0.63	31.35	$30K–$39K	Associate's	50.14
			(9.40)			(7.43)
Private	98	0.61	31.55	$30K–$39K	Associate's	48.91
			(9.74)			(6.46)
Private-Positive	94	0.62	31.45	$30K–$39K	Associate's	48.69
			(10.31)			(6.50)
Private-Zero	93	0.63	31.40	$40K–$49K	Associate's	49.37
			(8.13)			(6.15)
Pooled	383	0.62	31.44	$30K–$39K	Associate's	49.27
			(9.40)			(6.64)
Tests for differences among groups (p)		0.985^a^	0.999^b^	0.022^c^	0.545^c^	0.466^b^

a Chi-square goodness of fit.

b Analysis of variance.

c Kruskal-Wallis one-way analysis of variance.

**Figure 1:**
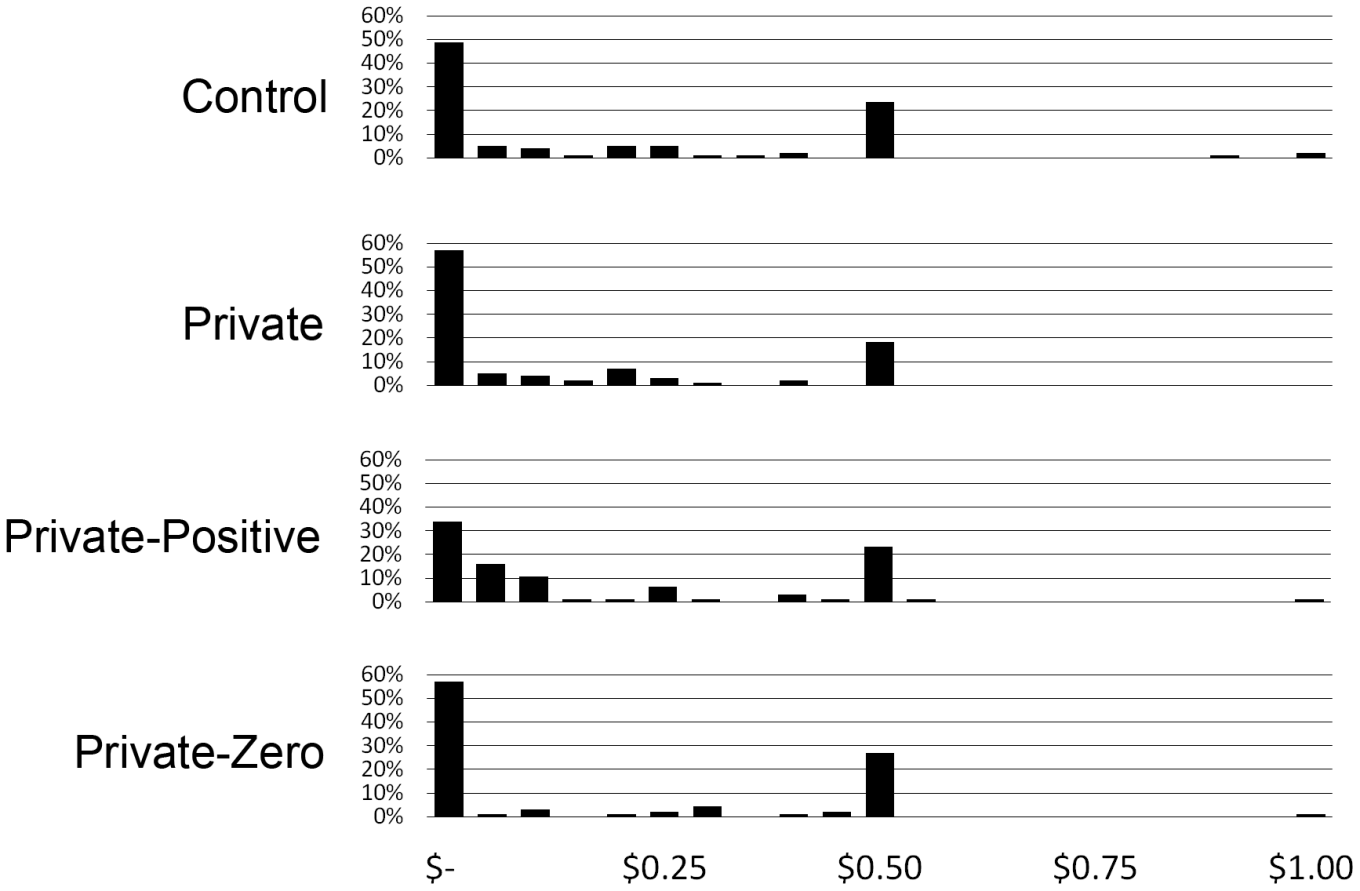
Distribution of Dictator donations across the four conditions.

Fig. 1 displays the distributions of donations across the four conditions. The mean donations did not significantly differ among the conditions (Table 2) (ANOVA, p = 0.227), although differences in their distributions approached significance (Kruskal-Wallis, p = 0.081). Overall levels of donations in the Private condition were lower than those in the Control condition, although the difference only attained one-tailed significance using a parametric t-test (p = 0.071), and was not significant using a non-parametric Mann-Whitney test (p = 0.140). H1 is thus tentatively supported. Participants in the Private-Positive group were less likely to donate zero (Χ^2^ = 4.404, p = 0.036) and more likely to donate the minimum non-zero amount (Χ^2^ = 6.059, p = 0.014), in support of H2.

**Table 2:** Dictator Game Donations.

	N	Mean (SD)	Median	Give 0 (%)	Give 5 (%)	Mean >0 (SD)	Median >0	Mean >5 (SD)	Median >5
Control	98	19.3 (¢)	5	48	5	37.8	50	41.4	50
		(24.9)		(49.0)	(5.1)	(22.7)		(21.0)	
Private	98	13.5†	0	56	5	31.4	27.5	35.0	40
		(19.5)		(57.1)	(5.1)	(18.0)		(16.1)	
Private-Zero	93	18.3	0	53	1	42.6	50	43.6	50
		(23.9)		(57.0)	(1.1)	(16.8)		(15.9)	
Private-Pos.	94	19.3	7.5	32*	15*	29.2*	25*	36.9	50
		(22.4)		(34.0)	(16.1)	(21.7)		(16.1)	
Tests for differences among groups (p)		0.227^a^	0.081^b^	0.105^c^	<0.001^c^	0.001^a^	0.002^b^	0.140^a^	0.178^b^

† p<0.10,

*p<0.05, Tests for difference from Control condition: t-test, Mann-Whitney U test, chi-square test used to test for differences in means, medians, and proportions respectively.

a Analysis of variance.

b Kruskal-Wallis one-way analysis of variance.

c Chi-square goodness of fit.

The remaining hypotheses received less support. The proportions of individuals donating zero were higher in the Private and Private-Zero conditions compared to the Control condition, in the directions predicted by H3a and H3b, but the differences were not significant (respectively, Χ^2^ = 1.311, p = 0.252; Χ^2^ = 1.229, p = 0.268). Participants in the Private condition who donated a non-zero amount did not donate significantly more than those in the Control condition (t = 1.469, p = .145; Mann-Whitney U = 905, p = 0.233), failing to support H2a, although the result was in the predicted direction. Similarly, participants in the Private-Positive condition who donated more than the minimum non-zero amount (5 cents), did not donate significantly more than those in the Control condition (t = 1.079, p = 0.283; Mann-Whitney U = 952.5, p = 0.381), failing to support H2b, although, again, the result was in the predicted direction.

## Discussion

The results suggest that making recipients aware of anonymous donor decisions impacts the psychological cost to those who donate zero, but has very minor, if any, impacts on actual donor behavior. This study replicates the results found by studies incorporating an option to exit the game for a minor cost [23]–[27]— participants in the Private-Positive condition were less likely to donate zero, and more likely to donate the minimum positive amount, in support of H2 (Table 3). It appears that this is indeed the impact of those who would have otherwise donated zero instead opting to donate five cents, as the reduction in zero donations is comparable to the augmentation in five-cent donations. Additionally, the fact that participants devised such a strategy independently suggests that this effect is not merely an experimenter demand effect induced by describing the exit strategy as it has been done in previous studies. This suggests that participants who donate zero do incur a psychological cost from their decision being revealed to the participant, which is at least greater than the cost of donating the minimum donation for some.

**Table 3:** Tests of Hypotheses.

Hypotheses	Predicted Direction?	Parametric p	Non-parametric p
H1: Lower donations in Private	Yes	0.140	0.071
H2: Fewer zero donations and more minimum positive donations in Private-Positive	Yes		0.036, 0.014
H3a: More zero donations in Private	Yes		0.252
H3b: More zero donations in Private-Positive	Yes		0.268
H4a: Non-zero donations will be smaller in Private	Yes	0.145	0.233
H4b: Donations greater than minimum positive will be smaller in Private Positive	Yes	0.283	0.381

Removing this psychological cost associated with revealing the decision to participants, however, does not appear to substantially motivate those who would typically donate a non-zero amount to be more likely to donate zero. Participants were not significantly more likely to donate zero in either the Private or Private-Zero conditions compared to the control. The lack of an effect between the Control and Private-Zero conditions is surprising, as the options presented to participants are similar to those presented to participants in previous studies who donated a non-zero amount and were then given the option to exit. Despite the finding that many such participants opted to exit in these studies, those in the current study did not independently decide to donate zero. Caution is necessary in declaring the null with confidence, however, as both tests result in non-significant effects in the predicted direction. Furthermore, if we pool the Private and Control conditions with those in the Dana et al. [23] study, the effect attains one tailed-significance (controlling for study, logistic regression, B = 0.440, p = 0.093).

Similarly, privacy did not appear to induce participants to give significantly lower non-zero donations. Participants who donated a non-zero amount in the Private condition and those who donated more than the minimum non-zero amount in the Private-Positive condition did not donate significantly more than their counterparts in the Control condition. Thus, the reporting of donor decisions to recipients does not appear to substantially induce participants to pursue prestige. Again, both effects were in the predicted direction. The effects were thus in the predicted directions for all four hypotheses relating to prestige and shame (H3a, H3b, H4a, and H4b), and the p-values, while not significant, might be interpreted in concert as suggestive. However, given the relatively large sample sizes used in this study, if these effects are real, they are likely very minor.

It's important to note that even the private conditions used in the current study might not elicit truly private behavior in an experimental context, as decisions must still be recorded, analyzed by the researcher, etc. Participants might therefore still be responding to cues of non-privacy, while truly private behaviors would trend even more towards selfishness. Alternatively, participants might be responding to experimenter demand effects in this study and lowering their donations because of the emphasis placed on contrived privacy rules in the instructions of the experiment. These caveats notwithstanding, there is strong evidence that the revealing of anonymous donor decisions to recipients increases the psychological cost (likely in the form of shame) that selfish-acting participants experience. Removing this cost appears to have a minor effect on overall donation levels, although the individual contributions to this effect of more individuals donating zero and those who donate a non-zero amount donating less were too minor to detect, even with sample sizes approaching 100.

## Supporting Information

S1 Fig **Research Instrument.**
(PDF)

S1 Table **Raw Data.**
(PDF)
