# Supplementary material for: Anonymity versus Privacy in the Dictator Game: Revealing Donor Decisions to Recipients Does Not Substantially Impact Donor Behavior
Source: PLoS One. 2014 Dec 22;9(12):e115419. doi: 10.1371/journal.pone.0115419 (PMC4274055; doi:10.1371/journal.pone.0115419)
Supplement: S1 Fig — Research Instrument. (PDF) [file pone.0115419.s001.pdf]

Consent & Information

You are being invited to take part in a research study being conducted by Texas A&M University and are asked to read this form so that you know about this research study. The information in this form is provided to help you decide whether or not to take part. If you decide to take part in the study, you will be asked to sign this consent form. If you decide you do not want to participate, there will be no penalty to you, and you will not lose any benefit you normally would have.

WHY IS THIS STUDY BEING DONE?
The purpose of this study is to learn about peoples social tendencies. This study will help us learn about human sociality.

WHY AM I BEING ASKED TO BE IN THIS STUDY?
You are being asked to be in this study because of your affiliation with Amazon's Mechanical Turk (MTurk) survey program.

HOW MANY PEOPLE WILL BE ASKED TO BE IN THIS STUDY?
400 people will take part in this study.

WHAT ARE THE ALTERNATIVES TO BEING IN THIS STUDY?
The alternative is not to participate.

WHAT WILL YOU BE ASKED TO DO IN THIS STUDY?
Your participation in this study will last up to 10 minutes once you start the survey. You will be asked to divide $1.00 between yourself and an anonymous recipient. You will also be asked to answer basic demographic questions and questions about your certain attitudes and behaviors.

ARE THERE ANY RISKS TO ME?
The things that you will be doing have no more/ risk than you would come across in everyday life. You do not have to answer anything you do not want to.

ARE THERE ANY BENEFITS TO ME?
There is no direct benefit to you by being in this study. What the researchers find out from this study will help society as a whole better understand how people behave socially.

WILL THERE BE ANY COSTS TO ME?
Aside from your time, there are no costs for taking part in the study.

WILL I BE PAID TO BE IN THIS STUDY?
You will receive $0.10 payment via the Amazon MTurk program. You will also be allowed to keep any of the $1.00 you allocate to yourself.

WILL INFORMATION FROM THIS STUDY BE KEPT PRIVATE?
The records of this study will be kept private. Your identity can not be linked to the research data, and no identifiers linking you to this study will be included in any sort of report that might be published. Research records will be stored securely and only Dr. Jeffrey Winking will have access to the records.

MTurk id's will be stored on a password protected computer until the end of the study at which time they will be

deleted.

Information about you will be kept confidential to the extent permitted or required by law. People who have access to your information include the Principal Investigator and research study personnel. Representatives of regulatory agencies such as the Office of Human Research Protections (OHRP) and entities such as the Texas A&M University Human Subjects Protection Program may access your records to make sure the study is being run correctly and that information is collected properly.

WHOM CAN I CONTACT FOR MORE INFORMATION?
You can call the Principal Investigator to tell him/her about a concern or complaint about this research study. The Principal Investigator is Dr. Jeffrey Winking and can be called at 979-845-5242 or emailed at jwinking@tamu.edu.

For questions about your rights as a research subject; or if you have questions, complaints, or concerns about the research and cannot reach the Principal Investigator or want to talk to someone other than the Investigator, you may call the Texas A&M Human Subjects Protection Program office.

- Phone number: (979) 458-4067
- Email: irb@tamu.edu

MAY I CHANGE MY MIND ABOUT PARTICIPATING?
You have the choice whether or not to be in this research study. You may decide to not begin or to stop the study at any time. If you choose not to be in this study, there will be no effect on you or your relationship with the Amazon MTurk program.

By participating in the survey, you are giving permission for the investigator to use your information for research purposes.

Thank you.
Jeffrey Winking

I understand that by checking this box, I am providing informed consent for this study.

- ○ I consent to participate.
- ○ I do not consent to participate

Thank you for your consideration. Please return the hit if you have already accepted it.

Please complete the following task and fill out the surveys that follow. At the end of the survey, you will be given a unique code to copy and paste into the original MTurk hit before submitting.

More than Zero Knowledge

For this task, you will have to decide how to split $1.00 between yourself and another MTurk participant who will be randomly paired with you. This individual will also receive a $0.10 reward for participating. (This will really happen--another MTurk participant will actually receive the amount you decide on.)

This individual will also fill out the surveys that you will fill out after this task. If you allocate more than 0 to this individual, he or she will then read about this study and how you decided to split the $1.00. Each of you will be given the appropriate amount as bonuses.

However, if you decide to keep all of the $1.00 and allocate nothing to this individual, he or she will NOT be told about this study or how you decided to split the $1.00, and only you will receive the bonus.

So, if you decide to allocate nothing to the other individual, he or she will never know your decision took place. If

you allocate anything other than zero, he or she will know about this decision and how much you allocated.

Even if the other individual reads of your decision, he or she will not be able to identify you or link your decision to you. It is completely confidential.

You will choose the amount you wish for the RECIPIENT to receive. You will then receive whatever is left.

Please answer the following questions to make sure that you understand the task. If you do not answer them correctly, you will be asked to reread the instructions and answer them again.

If you choose for the other individual to receive $0.00, what will happen?

I will receive a bonus of:

The other individual will:

- Know about this study and how I split the $1.00
- Not know about this study or how I split the $1.00

If you choose for the other individual to receive $0.25, what will happen?

I will receive a bonus of:

The other individual will:

- Know about this study and how I split the $1.00
- Not know about this study or how I split the $1.00

If you choose for the other individual to receive $0.75, what will happen?

I will receive a bonus of:

The other individual will:

- Know about this study and how I split the $1.00
- Not know about this study or how I split the $1.00

Please read the instructions again and answer the questions to check that you understand the the task. If you do

not answer them correctly, you will be asked to reread the instructions and answer them again.

For this task, you will have to decide how to split $1.00 between yourself and another MTurk participant who will be randomly paired with you. This individual will also receive a $0.10 reward for participating.

This individual will fill out the surveys as you have done. If you allocate more than 0 to this individual, he or she will then read about this study and how you decided to split the $1.00. Each of you will be given the appropriate amount as bonuses.

However, if you decide to keep all of the $1.00 and allocate nothing to this individual, he or she will NOT be told about this study or how you decided to split the $1.00, and only you will receive the bonus.

So, if you decide to allocate nothing to the other individual, he or she will never know your decision took place. If you allocate anything other than zero, he or she will know about this decision and how much you allocated.

Even if the other individual reads of your decision, he or she will not be able to identify you or link your decision to you. It is completely confidential.

You will choose the amount you wish for the RECIPIENT to receive. You will then receive whatever is left.

Please answer the following questions to make sure that you understand the task. If you do not answer them correctly, you will be asked to reread the instructions and answer them again.

If you choose for the other individual to receive $0.00, what will happen?

I will receive a bonus of:

The other individual will:

- Know about this study and how I split the $1.00
- Not know about this study or how I split the $1.00

If you choose for the other individual to receive $0.25, what will happen?

I will receive a bonus of:

The other individual will:

- Know about this study and how I split the $1.00
- Not know about this study or how I split the $1.00

If you choose for the other individual to receive $0.75, what will happen?

I will receive a bonus of:

The other individual will:

- Know about this study and how I split the $1.00
- Not know about this study or how I split the $1.00

Now for the real thing. You can split the $1.00 however you wish.

Remember, you will choose the amount you wish for the RECIPIENT to receive. You will then receive whatever is left.

How much of the $1.00 do you want the Recipient to receive? Remember, you will receive the remainder as a bonus.

Only Zero Knowledge

For this task, you will have to decide how to split $1.00 between yourself and another MTurk participant who will be randomly paired with you. This individual will also receive a $0.10 reward for participating. (This will really happen--another MTurk participant will actually receive the amount you decide on.)

This individual will also fill out the surveys that you will fill out after this task. If you allocate 0 to this individual, he or she will then read about this study and how you decided to split the $1.00. You will receive $1.00 as a bonus and the other individual will receive nothing as a bonus.

However, if you decide to allocate more than 0 to this individual, he or she will NOT be told about this study or how you decided to split the $1.00. After they complete the surveys, they will receive whatever you allocate as a bonus with a note that reads "Thanks! Here's a bonus of XXX", where the XXX will be what you allocate.

So, if you decide to allocate something other than zero, the other individual will never know your decision took place. If you allocate zero to the individual, he or she will know about this decision.

Even if the other individual reads of your decision, he or she will not be able to identify you or link your decision to you. It is completely confidential.

You will choose the amount you wish for the RECIPIENT to receive. You will then receive whatever is left.

Please answer the following questions to make sure that you understand the task. If you do not answer them correctly, you will be asked to reread the instructions and answer them again.

If you choose for the other individual to receive $0.00, what will happen?

I will receive a bonus of:

The other individual will:

- ◯ Know about this study and how I split the $1.00
- ◯ Not know about this study or how I split the $1.00

If you choose for the other individual to receive $0.25, what will happen?

I will receive a bonus of:

The other individual will:

- ◯ Know about this study and how I split the $1.00
- ◯ Not know about this study or how I split the $1.00

If you choose for the other individual to receive $0.75, what will happen?

I will receive a bonus of:

The other individual will:

- ◯ Know about this study and how I split the $1.00
- ◯ Not know about this study or how I split the $1.00

Please read the instructions again and answer the questions to check that you understand the the task. If you do not answer them correctly, you will be asked to reread the instructions and answer them again.

For this task, you will have to decide how to split $1.00 between yourself and another MTurk participant who will be randomly paired with you. This individual will also receive a $0.10 reward for participating.

This individual will fill out the surveys as you have done. If you allocate 0 to this individual, he or she will then read about this study and how you decided to split the $1.00. You will receive $1.00 as a bonus and the other individual will receive nothing as a bonus.

However, if you decide to allocate more than 0 to this individual, he or she will NOT be told about this study or how you decided to split the $1.00. After they complete the surveys, they will receive whatever you allocate as a bonus with a note that reads "Thanks! Here's a bonus of XXX", where the XXX will be what you allocate.

So, if you decide to allocate something other than zero, the other individual will never know your decision took place. If you allocate zero to the individual, he or she will know about this decision.

Even if the other individual reads of your decision, he or she will not be able to identify you or link your decision to you. It is completely confidential.

You will choose the amount you wish for the RECIPIENT to receive. You will then receive whatever is left.

Please answer the following questions to make sure that you understand the task. If you do not answer them correctly, you will be asked to reread the instructions and answer them again.

If you choose for the other individual to receive $0.00, what will happen?

I will receive a bonus of:

The other individual will:

- Know about this study and how I split the $1.00
- Not know about this study or how I split the $1.00

If you choose for the other individual to receive $0.25, what will happen?

I will receive a bonus of:

The other individual will:

- Know about this study and how I split the $1.00
- Not know about this study or how I split the $1.00

If you choose for the other individual to receive $0.75, what will happen?

I will receive a bonus of:

The other individual will:

- Know about this study and how I split the $1.00
- Not know about this study or how I split the $1.00

Now for the real thing. You can split the $1.00 however you want.

Remember, you will choose the amount you wish for the RECIPIENT to receive. You will then receive whatever is left.

How much of the $1.00 do you want the Recipient to receive? Remember, you will receive the remainder as a bonus.

▼

No Knowledge

For this task, you will have to decide how to split $1.00 between yourself and another MTurk participant who will be randomly paired with you. This individual will also receive a $0.10 reward for participating. (This will really happen--another MTurk participant will actually receive the amount you decide on.)

This individual will also fill out the surveys that you fill out after this task, but for this individual, the study will then end. He or she will NOT read anything about this portion of the study or how you decided to split the $1.00. After finishing, the individual will receive whatever you allocate as a bonus. If you allocate 0, the individual will not receive any bonus. If you allocate more than 0, the individual will receive a bonus with a note that reads "Thanks! Here's a bonus of XXX", where the XXX will be what you allocate.

So, regardless of how much you allocate to this individual, he or she will never know that this decision took place.

This individual will not be able to identify you or link your decision to you. It is completely confidential.

You will choose the amount you wish for the RECIPIENT to receive. You will then receive whatever is left.

Please answer the following questions to make sure that you understand the task. If you do not answer them correctly, you will be asked to reread the instructions and answer them again.

If you choose for the other individual to receive $0.00, what will happen?

I will receive a bonus of:

The other individual will:

- Know about this study and how I split the $1.00
- Not know about this study or how I split the $1.00

If you choose for the other individual to receive $0.25, what will happen?

I will receive a bonus of:

The other individual will:

- Know about this study and how I split the $1.00
- Not know about this study or how I split the $1.00

If you choose for the other individual to receive $0.75, what will happen?

I will receive a bonus of:

The other individual will:

- Know about this study and how I split the $1.00
- Not know about this study or how I split the $1.00

Please read the instructions again and answer the questions to check that you understand the the task. If you do not answer them correctly, you will be asked to reread the instructions and answer them again.

For this task, you will have to decide how to split $1.00 between yourself and another MTurk participant who will be randomly paired with you. This individual will also receive a $0.10 reward for participating.

He or she will fill out the surveys as you have done, but for this individual, the study will then end. He or she will NOT read anything about this portion of the study or how you decided to split the $1.00. After finishing, the individual will receive whatever you allocate as a bonus. If you allocate 0, the individual will not receive any bonus. If you allocate more than 0, the individual will receive a bonus with a note that reads "Thanks! Here's a bonus of XXX", where the XXX will be what you allocate.

So, regardless of how much you allocate to this individual, he or she will never know that this decision took place.

No one will be able to identify you or link your decision to you. It is completely confidential.

You will choose the amount you wish for the RECIPIENT to receive. You will then receive whatever is left.

Please answer the following questions to make sure that you understand the task. If you do not answer them correctly, you will be asked to reread the instructions and answer them again.

If you choose for the other individual to receive $0.00, what will happen?

I will receive:

The other individual will:

- Know about this study and how I split the $1.00
- Not know about this study or how I split the $1.00

If you choose for the other individual to receive $0.25, what will happen?

I will receive:

The other individual will:

- Know about this study and how I split the $1.00
- Not know about this study or how I split the $1.00

If you choose for the other individual to receive $0.75, what will happen?

I will receive:

The other individual will:

- Know about this study and how I split the $1.00
- Not know about this study or how I split the $1.00

Now for the real thing. You can split the $1.00 however you wish.

Remember, you will choose the amount you wish for the RECIPIENT to receive. You will then receive whatever is left.

How much of the $1.00 do you want the Recipient to receive? Remember, you will receive the remainder as a bonus.

▼

Survey

What is your gender?

- Male
- Female

What is your age?

What is your highest level of education?

▼

What is your annual household income?

- $0 - $10,000
- $10,000 - $19,999
- $20,000 - $29,999
- $30,000 - $39,999
- $40,000 - $49,999
- $50,000 - $59,999
- $60,000 - $69,999
- $70,000 - $79,999
- $80,000 - $89,999
- $90,000 - $99,999
- $100,000 - $124,999
- $125,000 - $149,999
- $150,000 - $199,999
- >$200,000

Please indicate whether you strongly agree, agree, neither agree nor disagree, disagree, or strongly disagree with the following statements:

	Strongly Disagree	Disagree	Neither Agree nor Disagree	Agree	Strongly Agree
People should be willing to help others who are less fortunate.					
Those in need have to learn to take care of themselves and not depend on others.					
Personally assisting people in trouble is very important to me.					
These days people need to look after themselves and not overly worry about others.					

During the past 12 months, how often have you done each of the following things:

	More than once a week	Once a week	Once a month	At least 2 or 3 times in the past year	Once in the past year	Not at all in the past year
Donated blood						
Given food or money to a homeless person						
Allowed a stranger to go ahead of you in line						
Done volunteer work for a charity						
Given money to a charity						
Offered your seat on a bus or in public place to a stranger who						

was standing						
Looked after a person's plants, mail, or pets while they were away	○	○	○	○	○	○
Carried a stranger's belongings, like groceries, a suitcase, or a shopping bag	○	○	○	○	○	○
Given directions to a stranger	○	○	○	○	○	○
Let someone you didn't know well borrow an item of some value like dishes or tools	○	○	○	○	○	○
Attended church or religious services	○	○	○	○	○	○

Debriefing

Thank you for participating!

This study is designed to explore which parts of experiments might cause people to act more charitably. Here, we're using a game called the Dictator Game, in which one person "dictates" how much to give to a recipient. We want to know if informing recipients of the dictator's actions, even when the dictator remains anonymous, affects the dictator's donations. This will help us learn about better ways to design future experiments. It will also teach us about the nature of humans' charitable motivations.

If you'd like to read about the results of this study, please check the following website in the future, which will link to any resulting publications.

http://anthropology.tamu.edu/html/profile--jeffreywinking.html

Thanks again, and please remember to copy the code on the next page into the original MTurk page.

Thank you,
Jeffrey Winking
